# Body temperature in the acute phase and clinical outcomes after acute ischemic stroke

**DOI:** 10.1371/journal.pone.0296639

**Published:** 2024-01-11

**Authors:** Satomi Mezuki, Ryu Matsuo, Fumi Irie, Yuji Shono, Takahiro Kuwashiro, Hiroshi Sugimori, Yoshinobu Wakisaka, Tetsuro Ago, Masahiro Kamouchi, Takanari Kitazono, on behalf of the Fukuoka Stroke Registry Investigators

**Affiliations:** 1 Department of Medicine and Clinical Science, Graduate School of Medical Sciences, Kyushu University, Fukuoka, Japan; 2 Emergency and Critical Care Center, Kyushu University Hospital, Fukuoka, Japan; 3 Department of Health Care Administration and Management, Graduate School of Medical Sciences, Kyushu University, Fukuoka, Japan; 4 Center for Cohort Study, Graduate School of Medical Sciences, Kyushu University, Fukuoka, Japan; 5 Division of Cerebrovascular Medicine and Neurology, Kyushu Medical Center, Fukuoka, Japan; Nicolaus Copernicus University in Torun: Uniwersytet Mikolaja Kopernika w Toruniu, POLAND

## Abstract

**Background:**

This study aimed to examine whether post-stroke early body temperature is associated with neurological damage in the acute phase and functional outcomes at three months.

**Methods:**

We included 7,177 patients with acute ischemic stroke within 24 h of onset. Axillary temperature was measured daily in the morning for seven days. Mean body temperature was grouped into five quintiles (Q1: 35.1‒36.5°C, Q2: 36.5‒36.7°C, Q3: 36.7‒36.8°C, Q4: 36.8‒37.1°C, and Q5: 37.1‒39.1°C). Clinical outcomes included neurological improvement during hospitalization and poor functional outcome (modified Rankin scale score, 3–6) at three months. A logistic regression analysis was performed to evaluate the association between body temperature and clinical outcomes.

**Results:**

The patient’s mean (SD) age was 70.6 (12.3) years, and 35.7% of patients were women. Mean body temperature was significantly associated with less neurological improvement from Q2 (odds ratios [95% confidence interval], 0.77 [0.65–0.99] vs. Q1) to Q5 (0.33 [0.28–0.40], P for trend <0.001) even after adjusting for potential confounders, including baseline neurological severity, C-reactive protein levels, and post-stroke acute infections. The multivariable-adjusted risk of poor functional outcome linearly increased from Q2 (1.36 [1.03–1.79]) to Q5 (6.44 [5.19–8.96], P for trend <0.001). These associations were maintained even in the analyses excluding patients with acute infectious diseases. Multivariable-adjusted risk of poor functional outcome was higher in patients with early body temperature elevation on days 1–3 and with longer duration with body temperature >37.0°C.

**Conclusions:**

Post-stroke early high body temperature is independently associated with unfavorable outcomes following acute ischemic stroke.

## Introduction

Body temperature (BT) is an important physical parameter of various biological responses. Proinflammatory cytokines, such as interleukin-1, interleukin-6, and tumor necrosis factor-α, are well-known pyrogens [1,2]. Because these proinflammatory cytokines are produced in various cell types, such as neurons, astrocytes, vascular cells, and microglia/macrophages, in the brain after acute ischemic stroke [2,3], BT is often elevated after acute ischemic stroke even without infectious complications. The extent of proinflammatory cytokine production is correlated largely to infarct volume [4] and neurological severity [5,6]; therefore, greater elevations in BT tend to occur in the acute phase in patients with more severe stroke with larger infarct volumes.

Many studies have examined the association between BT and clinical outcomes in patients with acute ischemic stroke. Most of them have suggested that patients with elevated BT are more likely to have poor functional outcomes, be discharged to nursing homes, or die after acute ischemic stroke [4,6–16]. Because high BT can enhance neuronal activity and release neurotransmitters [17,18], and increase metabolic demands [19], it may directly aggravate neuronal damage through glutamate-mediated excitotoxicity under ischemic conditions. However, patients with stroke often experience acute febrile illnesses, such as urinary and respiratory infections [20–23], which elevate BT and can worsen post-stroke outcomes. The increases in proinflammatory molecules under either infectious or non-infectious conditions may worsen functional recovery following an acute ischemic stroke while elevating BT [22,23]. In contrast, some studies have reported that elevated BT is associated with favorable functional outcomes in patients with acute ischemic stroke [24,25] after reperfusion therapy [26–28]. Adaptive BT elevation may be beneficial for thrombolysis [29] and macrophage function removing intra-infarct debris and leading to functional recovery [30]. Therefore, to assess whether high or elevated BT is an independent factor determining post-stroke functional outcomes, we should consider possible confounding factors, such as neurological severity, sterile- or non-sterile- inflammation, and reperfusion therapy, in addition to the timing and duration of BT elevation after acute ischemic stroke in large-scale analyses.

The present study aimed to clarify whether BT in the acute stage is independently associated with neurological course in the acute phase and functional outcomes at three months after ischemic stroke as a potential cause of unfavorable outcomes. We examined the poststroke trend in BT and sought to identify which BT parameter was more suitable for predicting poststroke functional outcomes.

## Materials and methods

### Data availability

Anonymized data are available from the corresponding author upon reasonable request.

### Study design

Data on patients with stroke were collected from the Fukuoka Stroke Registry (FSR), a multicenter, hospital-based prospective registry of patients with acute stroke [31,32]. The FSR comprises standardized and structured data from patients with stroke hospitalized in seven participating hospitals in Fukuoka Prefecture, Japan, within seven days of onset (UMIN Clinical Trial Registry, UMIN000000800). Details of the study design, data collection, and harmonization of the FSR have been previously described [31–34]. The participating hospitals were Kyushu University Hospital (Fukuoka, Japan), National Hospital Organization Kyushu Medical Center (Fukuoka, Japan), National Hospital Organization Fukuoka–Higashi Medical Center (Koga, Japan), Fukuoka Red Cross Hospital (Fukuoka, Japan), St. Mary’s Hospital (Kurume, Japan), Steel Memorial Yawata Hospital (Kitakyushu, Japan), and Japan Labour Health and Welfare Organization Kyushu Rosai Hospital (Kitakyushu, Japan). The study protocol was approved by the institutional review boards of all the participating hospitals. Written informed consent was obtained from all patients or their family members. Stroke was defined as the sudden onset of a non-convulsive and focal neurological deficit. Ischemic stroke was diagnosed by brain computed tomography, magnetic resonance imaging, or both.

### Participants

According to the FSR, 10,778 patients with acute ischemic stroke were hospitalized in the seven participating hospitals within 24 h of stroke onset between July 2007 and September 2019. To avoid the influence of pre-stroke functional dependency and pre-stroke febrile illness, we excluded 2,484 patients who were functionally dependent before stroke onset (modified Rankin Scale [mRS] score ≥2) and 357 patients with infection at stroke onset. We further excluded 604 patients with missing variables required for multivariable analysis and 156 patients who were lost to follow-up within three months after stroke onset. Finally, the data of 7,177 patients with acute ischemic stroke were included for analysis (S1 Fig).

### BT parameter

Axial temperature was measured on admission (day 1) and once daily in the morning for seven days after stroke onset. The mean BT values during the seven days after onset were calculated and grouped into five quintiles. The highest and lowest BT values during the seven days after onset were calculated to determine the BT values that are mostly associated with post-stroke outcomes.

### Clinical assessments

We evaluated the patients’ background characteristics, including age, sex, cardiovascular risk factors (hypertension, diabetes mellitus, dyslipidemia, and atrial fibrillation), history of stroke, body mass index, and estimated glomerular filtration rate. These variables have been defined in previous studies [31–34]. We assessed the onset-to-arrival time, defined as the time from stroke onset to hospital arrival; neurological severity; stroke subtype; reperfusion therapy; and acute infections. Baseline plasma C-reactive protein levels were measured on admission. Early hospital arrival was defined as time between stroke onset and hospital arrival equal to or less than 6 h. Neurological severity was assessed using the National Institutes of Health Stroke Scale (NIHSS). Ischemic stroke was classified into four subtypes based on etiology and according to the trial of ORG 10172 in acute stroke treatment criteria [35]: cardioembolism, large artery atherosclerosis, small vessel occlusion, and other etiologies (strokes due to other specific, undetermined, and unknown causes). Reperfusion therapy included intravenous thrombolysis with a recombinant tissue plasminogen activator and endovascular therapy with intra-arterial thrombolysis, endovascular thrombectomy, thromboaspiration, or angioplasty. Acute infections were defined as complications due to infectious diseases, including respiratory tract infections, urinary tract infections, sepsis, and other infections, developed during hospitalization for a stroke index.

### Study outcomes

The study outcomes included neurological courses (neurological improvement and worsening), poor functional outcomes, and death. A neurological improvement was defined as a ≥ 4-point decrease in NIHSS score during hospitalization or an NIHSS score of 0 at discharge. Neurological worsening was defined as a ≥ 2-point increase in NIHSS score at discharge compared with the NIHSS score on admission. Poor functional outcome was defined as a mRS score of 3–6. Death was defined as death from any cause. During hospitalization, trained stroke neurologists assessed the NIHSS and mRS scores. The mRS score at the three-month time point was evaluated by trained and certified research nurses, in person or through telephone assessment, using a standardized structured questionnaire validated in a previous study to minimize inter-rater variability [36].

### Statistical analysis

We compared baseline characteristics between the BT quintiles using analysis of variance, the Wilcoxon rank sum test, or the χ^2^ test and evaluated their trend according to the quintiles using Jonckheere–Terpstra or Cochran–Armitage tests as appropriate. We estimated the odds ratio (OR) and 95% confidence interval (CI) of the association between clinical outcomes and BT using a logistic regression analysis. The multivariable model included quantitative variables, such as age, body mass index, estimated glomerular filtration rate, baseline NIHSS score, and C-reactive protein levels, and categorical variables, such as sex, hypertension, dyslipidemia, diabetes mellitus, atrial fibrillation, previous stroke, early hospital arrival, stroke subtype, reperfusion therapy, and acute infections.

For subgroup analyses, the patients were divided into two subgroups according to age (<75 or ≥75 years), sex (women or men), neurological severity (severe stroke [NIHSS score ≥6] or non-severe stroke [NIHSS score <6]), stroke subtype (cardioembolism or non-cardioembolism), and reperfusion therapy. P for heterogeneity was calculated by adding the interaction term of the BT quintiles × subgroup variables to the model.

For sensitivity analysis, we analyzed the associations between BT and clinical outcomes in patients with<37.5°C BT in the acute stage and patients who did not develop acute infections during hospitalization for index stroke. Statistical analyses were performed using Stata 17 software (StataCorp LP, College Station, TX, USA). Statistical significance was set at p < 0.05.

## Results

### Baseline clinical characteristics

The mean age of the patients was 70.6±12.3 years, and 35.7% were women. Overall, following acute ischemic stroke, BT (median [interquartile range]°C) was elevated from day 1 (36.7 [36.5–37.0]°C) to day 2 (36.8 [36.6–37.2]°C), and gradually decreased to 36.6 [36.4–37.0]°C on day 7 (S2 Fig). The BT (median [interquartile range]°C) during the first seven days after stroke onset was 36.7 [36.5–37.0]°C.

Table 1 presents the differences in baseline characteristics according to the quintiles of the mean BT during the seven days after onset. Average age, NIHSS scores on admission, and C-reactive protein levels increased, while body mass index and estimated glomerular filtration rate decreased in higher mean BT quintiles. The frequency of female sex, atrial fibrillation, cardioembolism, early hospital arrival, and reperfusion therapy increased, while that of hypertension, dyslipidemia, and history of stroke decreased, in quintiles with higher mean BT levels. Post-stroke acute infections were more prevalent in quintiles with higher BT levels.

**Table 1:** Patient background characteristics according to the BT quintiles.

	Q1, n = 1475	Q2, n = 1506	Q3, n = 1326	Q4, n = 1446	Q5, n = 1424	
BT categories	(35.1– 36.5°C)	(36.5– 36.7°C)	(36.7– 36.8°C)	(36.8– 37.1°C)	(37.1– 39.1°C)	P_trend_
Age, years, mean ± SD	70.6 ± 10.5	69.9 ± 10.5	69.9 ± 12.1	69.7 ± 14.2	73.0 ± 13.5	<0.001
Women, n (%)	419 (28.4)	435 (28.9)	449 (33.9)	571 (39.5)	688 (48.3)	0.003
Risk factor, n (%)						
Hypertension	1143 (77.5)	1218 (80.9)	1086 (81.9)	1113 (77.0)	1129 (79.3)	0.002
Diabetes	441 (29.9)	432 (28.7)	397 (29.9)	397 (27.5)	389 (27.3)	0.67
Dyslipidemia	869 (58.9)	855 (56.8)	780 (58.8)	804 (55.6)	673 (47.3)	<0.001
Atrial fibrillation	344 (23.3)	311 (20.7)	257 (19.4)	332 (23.0)	569 (40.0)	<0.001
History of stroke, n (%)	233 (15.8)	238 (15.8)	203 (15.3)	198 (13.7)	184 (12.9)	0.83
BMI, kg/m^2^, mean ± SD	23.5 ± 3.2	23.4 ± 3.3	23.4 ± 3.7	23.1 ± 4.0	22.7 ± 4.0	<0.001
eGFR, mL/min/1.73m^2^, mean ± SD	68.5 ± 20.6	69.3 ± 22.0	67.9 ± 25.4	68.8 ± 26.5	66.9 ± 25.4	0.02
Early hospital arrival, n (%)	771 (52.3)	781 (51.9)	673 (50.8)	746 (51.6)	825 (57.9)	0.008
NIHSS score on admission, median (IQR)	0 (2–3)	1 (2–4)	1 (2–4)	1 (3–7)	3 (9–18)	<0.001
Stroke subtype, n (%)						
Cardioembolism	273 (18.5)	274 (18.2)	238 (17.9)	320 (22.1)	549 (38.6)	<0.001
Large artery atherosclerosis	156 (10.6)	212 (14.1)	158 (11.9)	232 (16.0)	263 (18.5)	
Small-vessel occlusion	422 (28.6)	468 (31.1)	457 (34.5)	417 (28.8)	197 (13.8)	
Others	624 (42.3)	552 (36.7)	473 (35.7)	477 (33.0)	415 (29.1)	
Reperfusion therapy, n (%)	134 (9.1)	131 (8.7)	160 (12.1)	248 (17.2)	435 (30.5)	<0.001
Intravenous thrombolysis	131 (8.9)	127 (8.4)	145 (10.9)	219 (15.1)	340 (23.9)	<0.001
Endovascular therapy	12 (0.8)	20 (1.3)	35 (2.6)	71 (4.9)	215 (15.1)	<0.001
Acute infection, n (%)	21 (1.4)	43 (2.9)	62 (4.7)	151 (10.4)	524 (36.8)	<0.001
C-reactive protein, mg/dL, median (IQR)	0.00 (0.07–0.20)	0.02 (0.10–0.21)	0.02 (0.10–0.25)	0.04 (0.10–0.30)	0.05 (0.14–0.48)	<0.001

BT, body temperature; P_trend_, P value for trend; SD, standard deviation; BMI, body mass index; eGFR, estimated glomerular filtration rate; NIHSS, National Institutes of Health Stroke Scale; IQR, interquartile range. Q1–Q5 indicate quintiles of mean BT (°C) during the first seven days after onset.

### Association between BT and neurological courses in the acute phase

**Fig 1:**
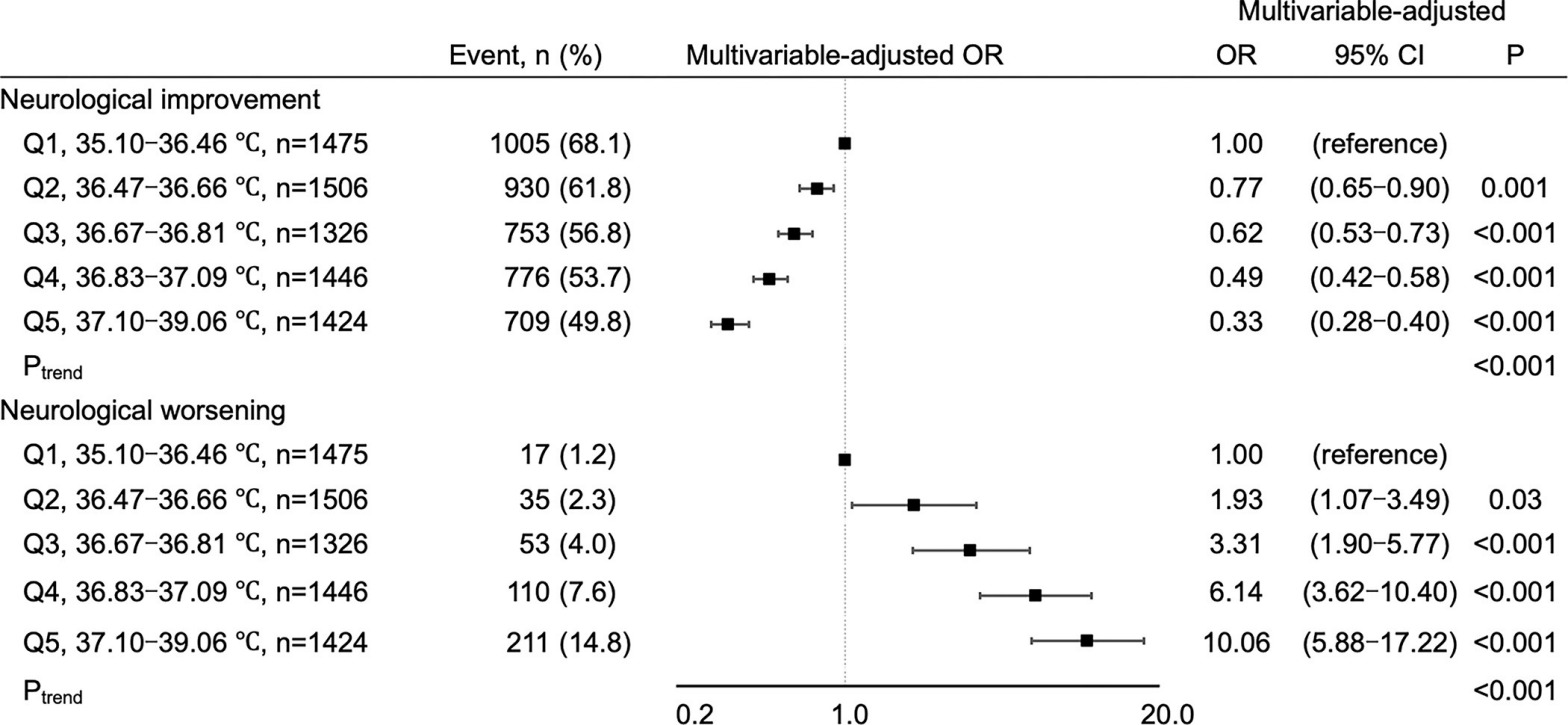
Association between BT and neurological courses. BT: body temperature, CI: confidence interval. Multivariable-adjusted odds ratios (square) and 95% confidence intervals (horizontal bars) of neurological courses in acute phase are shown for Q2-Q5 compared to Q1. The multivariable model included the following covariates: age, sex, hypertension, diabetes mellitus, dyslipidemia, atrial fibrillation, previous stroke, body mass index, estimated glomerular filtration rate, early hospital arrival, National Institutes of Health Stroke Scale score on admission, stroke subtype, reperfusion therapy, acute infections, and C-reactive protein level.

We first examined the association between acute-phase BT and neurological courses during hospitalization. The frequency of neurological improvement decreased, while that of neurological worsening increased in quintiles with higher mean BT (Fig 1). Multivariable analysis demonstrated that higher mean BT was significantly associated with less neurological improvement and neurological worsening, even after adjusting for initial neurological severity, post-stroke acute infections, and C-reactive protein levels (Fig 1).

### Association between acute-phase BT and clinical outcomes at three months

**Fig 2:**
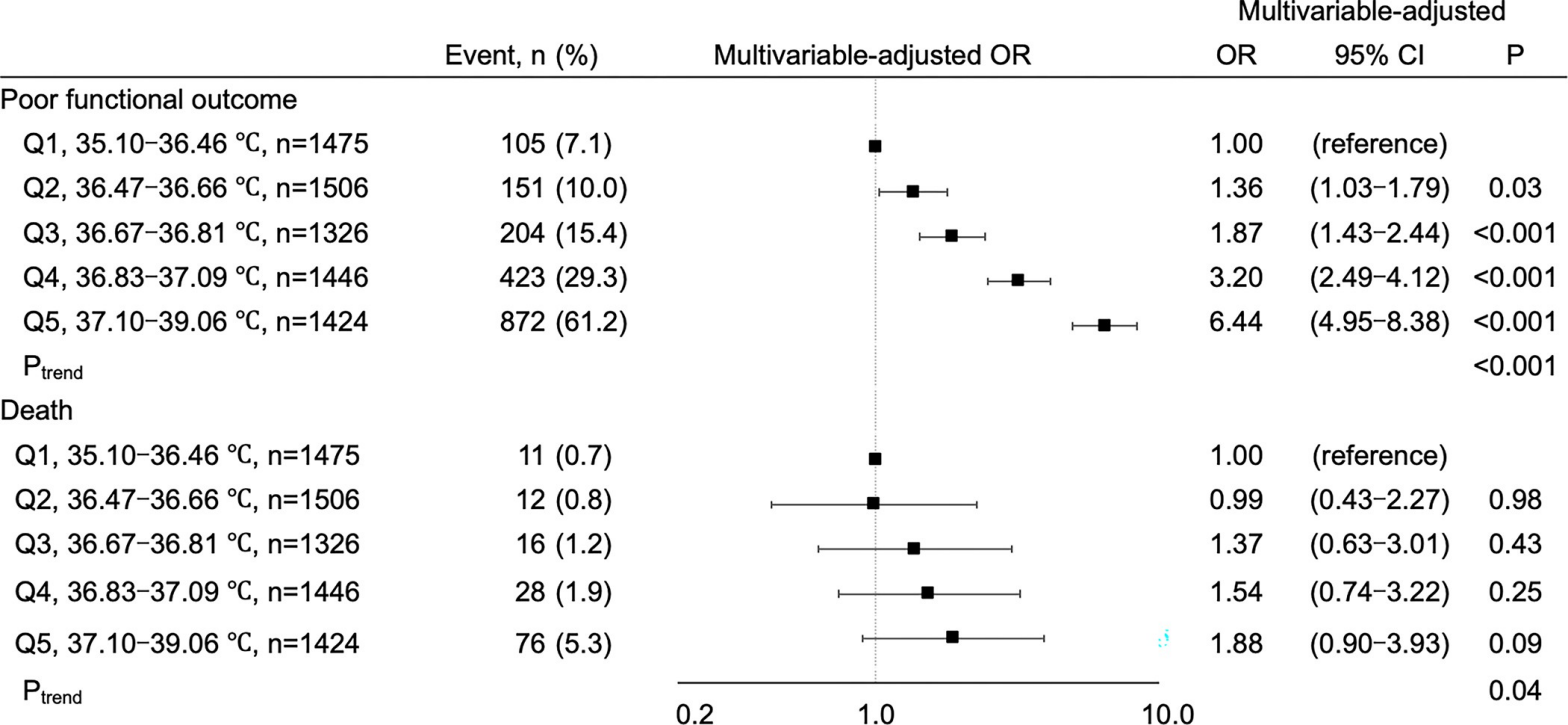
Association between BT and clinical outcomes at 3 months. BT: Body temperature, CI: Confidence interval. Multivariable-adjusted odds ratios (square) and 95% confidence intervals (horizontal bars) of clinical outcome at 3 months are shown for Q2-Q5 compared to Q1. The multivariable model included the following covariates: Age, sex, hypertension, diabetes mellitus, dyslipidemia, atrial fibrillation, previous stroke, body mass index, estimated glomerular filtration rate, early hospital arrival, National Institutes of Health Stroke Scale score on admission, stroke subtype, reperfusion therapy, acute infections, and C-reactive protein level.

We next examined the association between acute phase BT and clinical outcomes at three months. The frequency of poor functional outcome was higher in quintiles with higher BT in the acute phase (Fig 2). Multivariable analysis adjusting for confounders, including initial neurological severity, post-stroke acute infections, and C-reactive protein levels demonstrated that OR of poor functional outcome at three months increased according to the elevation of mean BT (Fig 2). No association between mean BT and risk of all-cause death was found at three months (Fig 2) or at discharge (S1 Table).

**Fig 3:**
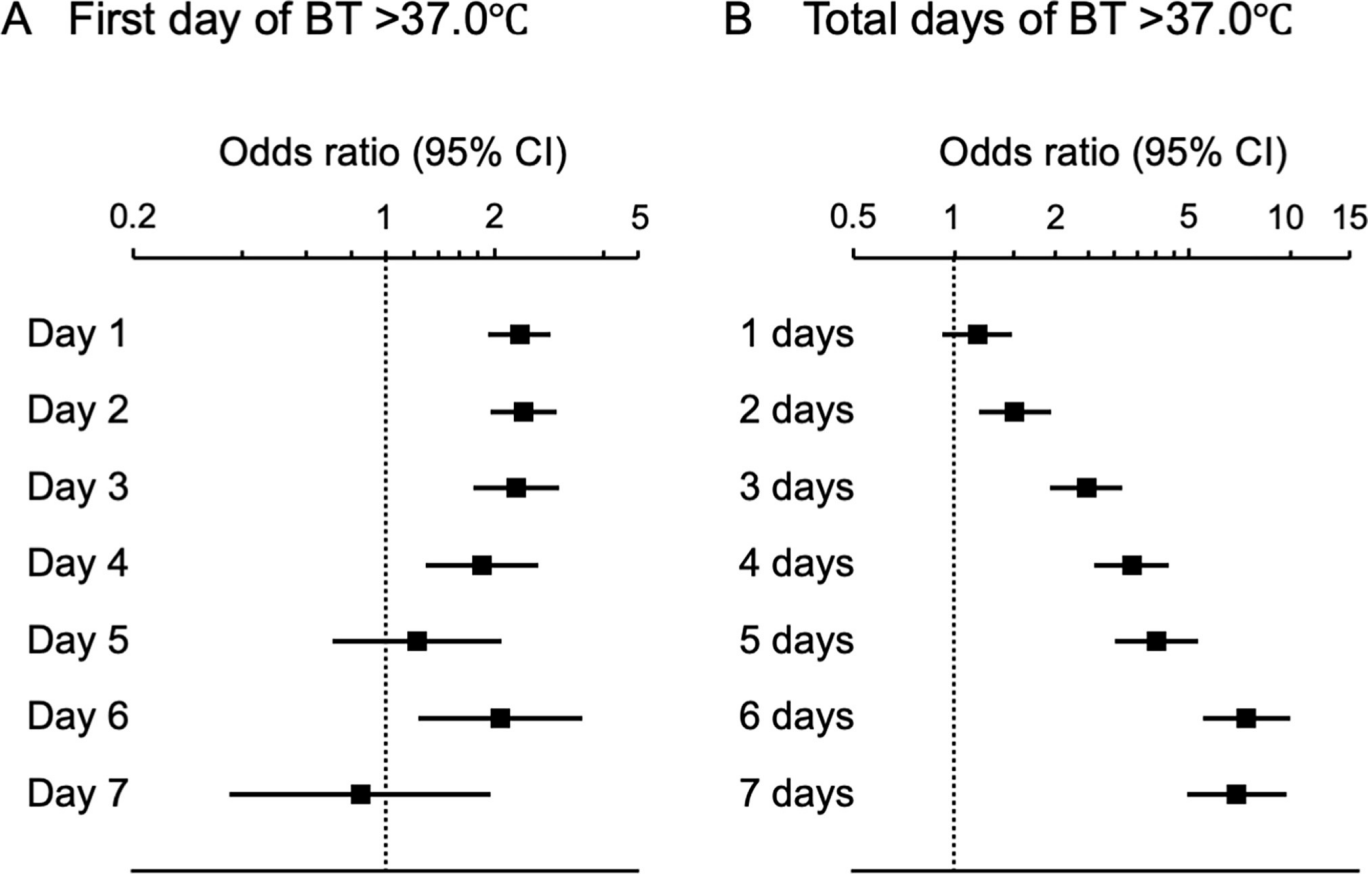
Associations between the duration of BT and poor functional outcome. BT: Body temperature, CI: Confidence interval. Multivariable-adjusted odds ratios (square) and 95% confidence intervals (horizontal bars) of poor functional outcome are shown for BT >37.0°C on each day when BT first exceeded 37.0°C (compared to BT ≤37.0°C on respective days, A), and total days of BT of >37.0°C (compared to BT of ≤37.0°C for 7 days after stroke onset, B). The multivariable model included the following covariates: Age, sex, hypertension, diabetes mellitus, dyslipidemia, atrial fibrillation, previous stroke, body mass index, estimated glomerular filtration rate, early hospital arrival, National Institutes of Health Stroke Scale score on admission, stroke subtype, reperfusion therapy, acute infections, and C-reactive protein level.

We then examined the association between the timing and duration of BT elevation and functional outcomes at three months. Regarding the timing of BT elevation, patients presenting with BT >37.0°C until day 3 had equally higher risks of poor functional outcome at three months, compared to those with BT ≤37.0°C, while the risk appeared to decrease in patients presenting with BT >37.0°C no later than day 4 (Fig 3A). The risk of poor functional outcomes increased proportionally to the duration of BT >37.0°C during the first seven days after stroke onset (Fig 3B).

We further examined the utility of the highest or lowest BT during the first seven days, instead of mean BT, as a parameter of BT for the association with functional outcomes. Although all three parameters showed similar trends of significant association with the outcomes, the highest ORs were obtained when mean BT was used (Table 2).

**Table 2:** Association between the lowest, mean, or highest BT and neurological courses and poor functional outcome.

		Age- and sex-adjusted		Multivariable-adjusted	
	Events/total	OR	P	OR	P
	(%)	(95% CI)		(95% CI)	
Neurological improvement					
Lowest BT	4173/7177	0.80		0.72	
	(58.1)	(0.77–0.84)	<0.001	(0.68–0.77)	<0.001
Mean BT	4173/7177	0.75		0.66	
	(58.1)	(0.71–0.79)	<0.001	(0.62–0.70)	<0.001
Highest BT	4173/7177	0.79		0.79	
	(58.1)	(0.76–0.83)	<0.001	(0.75–0.83)	<0.001
Neurological worsening					
Lowest BT	426/7177	1.86		1.53	
	(5.9)	(1.71–2.02)	<0.001	(1.38–1.70)	<0.001
Mean BT	426/7177	2.18		2.06	
	(5.9)	(1.99–2.38)	<0.001	(1.82–2.33)	<0.001
Highest BT	426/7177	1.95		1.62	
	(5.9)	(1.75–2.17)	<0.001	(1.43–1.83)	<0.001
Poor functional outcome					
Lowest BT	1755/7177	2.68		1.61	
	(24.5)	(2.51–2.86)	<0.001	(1.48–1.74)	<0.001
Mean BT	1755/7177	3.50		2.09	
	(24.5)	(3.24–3.79)	<0.001	(1.91–2.29)	<0.001
Highest BT	1755/7177	2.34		1.60	
	(24.5)	(2.18–2.52)	<0.001	(1.47–1.73)	<0.001

BT, body temperature; OR, odds ratio; CI, confidence interval.

Odds ratios of clinical outcomes per 1 SD of the lowest, mean, or highest BT during the first seven days after onset are shown.

The multivariable model included the following covariates: age, sex, hypertension, diabetes mellitus, dyslipidemia, atrial fibrillation, previous stroke, body mass index, estimated glomerular filtration rate, early hospital arrival, National Institutes of Health Stroke Scale score on admission, stroke subtype, reperfusion therapy, acute infections, and C-reactive protein level.

### Subgroup analysis

Subgroup analyses were performed to determine whether specific patients were susceptible to high BT. However, no heterogeneity was found in the associations between the mean BT and neurological improvement (S3 Fig) or poor functional outcome (S4 Fig) according to age, sex, neurological severity, stroke subtype, and reperfusion therapy.

### Sensitivity analysis

In this study, 813 patients developed acute infectious diseases (481 respiratory tract infections, 261 urinary tract infections, 33 sepsis, and 172 other infections) during hospitalization. We, therefore, performed sensitivity analysis after excluding the 813 patients and found that the significant association between BT elevation and acute-phase neurological courses and functional outcome at three months was maintained even in patients without acute infections (S2 Table). Moreover, similar trends were found in patients with BT<37.5°C throughout the seven days after stroke onset (S3 Table).

## Discussion

The major findings of this study are as follows: 1) according to the analyses using the quintiles of mean BT during the first seven days after acute ischemic stroke, higher BT was significantly associated with poor acute neurological outcomes (assessed by neurological improvement or worsening) and poor functional outcomes at three months, even after adjusting for confounders including neurological severity, C-reactive protein levels, and post-stroke acute infections. 2) The association was maintained irrespective of age, sex, neurological severity, stroke subtype, and reperfusion therapy. 3) The association was also maintained in sensitive analyses excluding patients with BT≥37.5°C or acute infections. 4) Multivariable-adjusted risk of poor functional outcome was higher in the groups with an earlier elevation of BT (day 1 to 3) or with a longer duration of BT >37.0°C during the first seven days.

### Changes in body temperature after acute ischemic stroke

BT appeared to be elevated from day 1, reached the peak on day 2, and gradually declined at least over seven days, although its variation range was not so large (< 0.5°C). We could not know the baseline (pre-stroke) BT in the patients; however, because endogenous pyrogens, such as the proinflammatory cytokine interleukin-6, are locally produced in ischemic areas [3], the BT elevation in the early days may reflect the extent of locally produced pyrogens or excessive biological response to the pyrogens, even without acute infectious complications. Presumably, the amounts of locally produced pyrogens may not be so large to induce a high-grade fever. Post-stroke BT elevation or higher basal BT based on basic metabolism was significantly associated with post-stoke functional outcomes.

We used three different BT parameters, *i*.*e*., mean BT, highest BT, and lowest BT, during the first seven days to examine the association between BT and post-stroke functional outcomes. The three BT parameters showed similar association trends with the outcomes, with the best OR observed for mean BT. This finding suggests that patients who experienced a longer duration of BT above 37°C during the first seven days had a higher likelihood of poor functional outcomes [4,5,37]. Therefore, we primarily used the mean BT in this study.

### Association between BT and clinical courses in the acute phase of ischemic stroke

The different handling of adjustment factors among previous studies may be a major cause of inconsistent results regarding the association between BT and poststroke functional outcomes [6,8,9,12–15,24,25]. Therefore, we included possible confounders that can affect BT, such as stroke subtypes, neurological severity [6,38], CRP as an inflammatory factor [39], and acute infectious complications [23] in this study. Even in the multivariable analysis, we found that higher BT in the acute phase was significantly associated with neurological worsening and poor neurological improvement. We also confirmed this by a subgroup analysis considering neurological severity and by sensitive analyses excluding patients with acute infectious diseases or those with BT≥37.5°C.

Some reports have suggested that higher BT may rather be associated with a better clinical course, particularly in patients undergoing reperfusion therapy either by intravenous thrombolysis [26–28,40] or endovascular thrombectomy [16,41]. However, the present subgroup analysis in patients undergoing reperfusion therapy showed an association between high BT and poor neurological improvement and neurological worsening. Average BT on day 1 (i.e., at the time of reperfusion therapy) was relatively high (36.8°C) in this study; therefore, this study may not include significant numbers of patients with low BT that can impair thrombolytic activity [29]. Because high BT can enhance the release of neurotransmitters [17,18] and increase metabolic demands [19], higher BT may directly aggravate neuronal damages in the very acute phase through glutamate-mediated excitotoxicity under ischemic conditions, regardless of reperfusion therapies.

### Association between acute phase BT and functional outcomes at three months

Higher BT, particularly on days 1–3, was associated not only with worse clinical courses in the acute phase but also with poor functional outcomes at three months without affecting mortality. Proinflammatory cytokines produced immediately after stroke onset in the brain and their downstream molecules, such as CRP [42,43], are apparently associated with enhanced neurovascular damage and poor clinical courses in the acute phase [3,44]; however, recent basic studies elucidate that these inflammatory molecules are indispensable for post-stroke restoration of blood flow and tissue repair leading to functional recovery [2,3,42,45]. Nevertheless, poststroke early increase in CRP has been found to be independently associated with poor functional recovery at three months [33,46]. Although we cannot completely exclude a link between BT and inflammatory factors unrelated to CRP, the present multivariable analyses, including CRP and coexisting infectious complications, highly suggests that BT in the acute phase is associated independently with functional outcomes at three months. Poor clinical courses associated with higher BT in the acute phase may explain the poor functional outcomes at three months.

In the clinical setting, therapeutic hypothermia has been accepted as an effective treatment after cardiac arrest [47] and in neonates with hypoxic-ischemic encephalopathy [48]. However, despite the experimental success of hypothermia in animal stroke models [49], there is limited evidence that therapeutic hypothermia or lowering BT using acetaminophen reduces mortality or improves functional outcomes in randomized control trials [50,51]. It remains unclear whether BT itself has a causal relationship to clinical outcomes after ischemic stroke. Nevertheless, we postulate the existence of specific factors that can induce an early excessive biological response leading to BT elevation and hindering functional recovery during the subacute phases, such as macrophage dysfunction [52].

### Study strengths and limitations

This study has some strengths. This study was conducted prospectively using a standardized method and involving a large number of patients. BT was measured daily, and clinical variables and inflammatory markers were collected in a standardized manner. Our study has some limitations that should be considered. First, although axillary temperature was measured once a day, frequent measurements of BT are needed to accurately assess the burden of BT and detect variability (fluctuation). Second, since the core body temperature is usually higher than the axillary temperature, BT in this study may be lower than that in other studies using the rectal or tympanic temperature. Third, baseline BT before stroke onset could neither be measured nor controlled for in this study. Therefore, we cannot determine whether the width of BT elevation following an ischemic stroke or high BT based on basic metabolism was associated with poor functional outcomes. Fourth, antipyretic treatments for BT were not evaluated. Fifth, we restricted study patients to patients without functional dependency before stroke onset. Therefore, it remains unknown whether the results can be also applied to patients who are functionally dependent before onset. Finally, since this study was performed in participating hospitals in the FSR in Japan and basic metabolism maintaining BT and biological responses elevating BT may differ among races due to genetic polymorphisms, the generalizability of the findings should be further validated in other cohorts.

## Conclusions

Higher BT in the acute stage was associated with both acute phase clinical courses and unfavorable functional outcomes at three months, independent of initial neurological severity, the inflammatory parameter CRP, and acute infections, and regardless of age, sex, and reperfusion therapy. Further studies are warranted to elucidate the clinical relevance of post-stroke BT and its management after acute ischemic stroke in clinical practice.

## Supporting information

S1 Fig Low chart of patient selection. (PDF)

S2 Fig Time course change of BT within 7 days after stroke onset. BT: body temperature. The median (circle) and the interquartile range between the first and third quartiles (box) of body temperature are shown against the days after stroke onset using a violin plot indicating the volume of the samples at each point by width.
(PDF)

S3 Fig Subgroup analysis of the association between BT and neurological improvement. OR: odds ratio, CI: confidence interval. OR (square) and 95% CI (horizontal bars) of neurological improvement are shown for Q1–Q4 compared to Q1 according to age (<75 y and ≥75 y), sex, stroke severity (National Institutes of Health Stroke Scale [NIHSS] score on admission: <6 and ≥6), stroke subtype (cardioembolism and non-cardioembolism), and reperfusion therapy. The multivariable model included the following covariates: age, sex, hypertension, diabetes mellitus, dyslipidemia, atrial fibrillation, previous stroke, body mass index, estimated glomerular filtration rate, early hospital arrival, National Institutes of Health Stroke Scale score on admission, stroke subtype, reperfusion therapy, acute infections, and C-reactive protein level.
(PDF)

S4 Fig Subgroup analysis of the association between BT and poor functional outcome. OR: odds ratio, CI: confidence interval. OR (square) and 95% CI (horizontal bars) of poor functional outcome are shown for Q1–Q4 compared to Q1 according to age (<75 y and ≥75 y), sex, stroke severity (National Institutes of Health Stroke Scale [NIHSS] score on admission: <6 and ≥6), stroke subtype (cardioembolism and non-cardioembolism), and reperfusion therapy. The multivariable model included the following covariates: age, sex, hypertension, diabetes mellitus, dyslipidemia, atrial fibrillation, previous stroke, body mass index, estimated glomerular filtration rate, early hospital arrival, National Institutes of Health Stroke Scale score on admission, stroke subtype, reperfusion therapy, acute infections, and C-reactive protein level.
(PDF)

S1 Table Association between BT and clinical outcomes at discharge. BT, body temperature; OR, odds ratio; CI, confidence interval; P_trend_, P-value for trend. Q1–Q5 indicate quintiles of mean BT (°C) during the first 7 days after stroke onset. The multivariable model included the following covariates: age, sex, hypertension, diabetes mellitus, dyslipidemia, atrial fibrillation, previous stroke, body mass index, estimated glomerular filtration rate, early hospital arrival, National Institutes of Health Stroke Scale score on admission, stroke subtype, reperfusion therapy, acute infections, and C-reactive protein level.
(PDF)

S2 Table Association between BT and neurological courses and poor functional outcome in patients without acute infections. BT, body temperature; OR, odds ratio; CI, confidence interval; P_trend_, P-value for trend. Q1–Q5 indicate quintiles of mean BT (°C) during the first 7 days after onset. The multivariable model included the following covariates: age, sex, hypertension, diabetes mellitus, dyslipidaemia, atrial fibrillation, previous stroke, body mass index, estimated glomerular filtration rate, early hospital arrival, National Institutes of Health Stroke Scale score on admission, stroke subtype, reperfusion therapy, acute infections, and C-reactive protein level.
(PDF)

S3 Table Association between BT and neurological courses and poor functional outcome in patients with BT <37.5°C. BT, body temperature; OR, odds ratio; CI, confidence interval; P_trend_, P-value for trend. Q1–Q5 quintiles of mean BT (°C) during the first 7 days after onset. The multivariable model included the following covariates: age, sex, hypertension, diabetes mellitus, dyslipidaemia, atrial fibrillation, previous stroke, body mass index, estimated glomerular filtration rate, early hospital arrival, National Institutes of Health Stroke Scale score on admission, stroke subtype, reperfusion therapy, acute infections, and C-reactive protein level.
(PDF)
