# Supplementary figures and images for: Body temperature in the acute phase and clinical outcomes after acute ischemic stroke
Source: PLoS One. 2024 Jan 11;19(1):e0296639. doi: 10.1371/journal.pone.0296639 (PMC10783745; doi:10.1371/journal.pone.0296639)

**S1 Figure. Flow chart of patient selection**

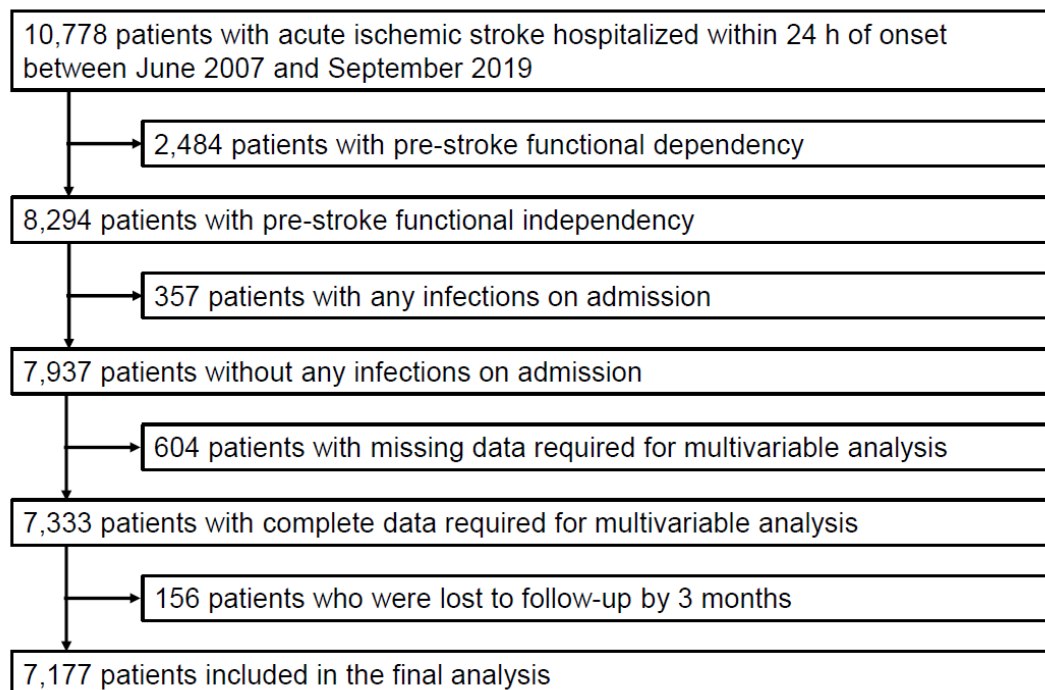

Supplement: S1 Fig — (PDF) [file pone.0296639.s001.pdf]
