# Supplementary material for: Body temperature in the acute phase and clinical outcomes after acute ischemic stroke
Source: PLoS One. 2024 Jan 11;19(1):e0296639. doi: 10.1371/journal.pone.0296639 (PMC10783745; doi:10.1371/journal.pone.0296639)
Supplement: S2 Fig — BT: body temperature. The median (circle) and the interquartile range between the first and third quartiles (box) of body temperature are shown against the days after stroke onset using a violin plot indicating the volume of the samples at each point by width. (PDF) [file pone.0296639.s002.pdf]

S2 Figure. Time course change in BT within 7 days after stroke onset

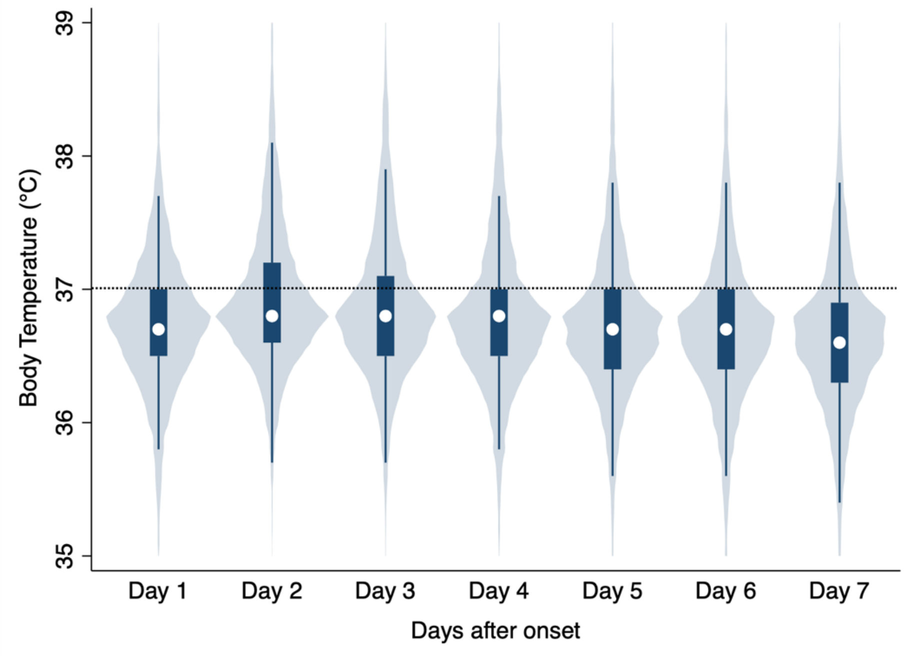

BT: body temperature.
The median (circle) and the interquartile range between the first and third quartiles (box) of body temperature are shown against the days after stroke onset using a violin plot indicating the volume of the samples at each point by width.
