# Supplementary material for: Body temperature in the acute phase and clinical outcomes after acute ischemic stroke
Source: PLoS One. 2024 Jan 11;19(1):e0296639. doi: 10.1371/journal.pone.0296639 (PMC10783745; doi:10.1371/journal.pone.0296639)
Supplement: S3 Fig — OR: odds ratio, CI: confidence interval. OR (square) and 95% CI (horizontal bars) of neurological improvement are shown for Q1–Q4 compared to Q1 according to age (<75 y and ≥75 y), sex, stroke severity (National Institutes of Health Stroke Scale [NIHSS] score on admission: <6 and ≥6), stroke subtype (cardioembolism and non-cardioembolism), and reperfusion therapy. The multivariable model included the following covariates: age, sex, hypertension, diabetes mellitus, dyslipidemia, atrial fibrillation, previous stroke, body mass index, estimated glomerular filtration rate, early hospital arrival, National Institutes of Health Stroke Scale score on admission, stroke subtype, reperfusion therapy, acute infections, and C-reactive protein level. (PDF) [file pone.0296639.s003.pdf]

S3 Figure. Subgroup analysis for the association between BT and neurological improvement

			OR	95%CI	P	P_h
Age < 75y						
Q1	618/917	(67.4)	1.00	(reference)		
Q2	592/974	(60.8)	0.77	(0.63-0.94)	0.01	
Q3	456/799	(57.1)	0.66	(0.54-0.81)	<0.001	
Q4	446/815	(54.7)	0.53	(0.43-0.65)	<0.001	
Q5	356/663	(53.7)	0.38	(0.30-0.49)	<0.001	
Age ≥ 75y						0.39
Q1	387/558	(69.4)	1.00	(reference)		
Q2	338/532	(63.5)	0.78	(0.60-1.02)	0.07	
Q3	297/527	(56.4)	0.58	(0.45-0.75)	<0.001	
Q4	330/631	(52.3)	0.47	(0.36-0.61)	<0.001	
Q5	353/761	(46.4)	0.31	(0.23-0.41)	<0.001	
Women						
Q1	304/419	(72.6)	1.00	(reference)		
Q2	271/435	(62.3)	0.64	(0.48-0.87)	0.005	
Q3	276/449	(61.5)	0.63	(0.47-0.86)	0.003	
Q4	311/571	(54.5)	0.42	(0.32-0.56)	<0.001	
Q5	343/688	(49.9)	0.28	(0.21-0.38)	<0.001	
Men						0.26
Q1	701/1056	(66.4)	1.00	(reference)		
Q2	659/1071	(61.5)	0.82	(0.68-0.99)	0.04	
Q3	477/877	(54.4)	0.60	(0.49-0.73)	<0.001	
Q4	465/875	(53.1)	0.52	(0.43-0.64)	<0.001	
Q5	366/736	(49.7)	0.37	(0.29-0.47)	<0.001	
Non-severe						
Q1	872/1313	(66.4)	1.00	(reference)		
Q2	780/1315	(59.3)	0.76	(0.64-0.90)	0.001	
Q3	565/1087	(52.0)	0.58	(0.49-0.69)	<0.001	
Q4	467/988	(47.3)	0.47	(0.39-0.56)	<0.001	
Q5	194/527	(36.8)	0.31	(0.24-0.39)	<0.001	
Severe						0.76
Q1	133/162	(82.1)	1.00	(reference)		
Q2	150/191	(78.5)	0.84	(0.48-1.47)	0.54	
Q3	188/239	(78.7)	0.80	(0.47-1.37)	0.42	
Q4	309/458	(67.5)	0.50	(0.31-0.80)	0.004	
Q5	515/897	(57.4)	0.33	(0.20-0.52)	<0.001	
Non-cardioembolism						
Q1	798/1202	(66.4)	1.00	(reference)		
Q2	744/1232	(60.4)	0.78	(0.66-0.92)	<0.001	
Q3	605/1088	(55.6)	0.63	(0.53-0.75)	<0.001	
Q4	565/1126	(50.2)	0.49	(0.41-0.58)	<0.001	
Q5	415/875	(47.4)	0.44	(0.35-0.54)	<0.001	
Cardioembolism						0.09
Q1	207/273	(75.8)	1.00	(reference)		
Q2	186/274	(67.9)	0.65	(0.44-0.95)	0.03	
Q3	148/238	(62.2)	0.46	(0.31-0.69)	<0.001	
Q4	211/320	(65.9)	0.47	(0.32-0.70)	<0.001	
Q5	294/549	(53.6)	0.21	(0.14-0.31)	<0.001	
No reperfusion						
Q1	897/1341	(66.9)	1.00	(reference)		
Q2	827/1375	(60.1)	0.76	(0.65-0.90)	0.001	
Q3	622/1166	(53.3)	0.60	(0.50-0.71)	<0.001	
Q4	600/1198	(50.1)	0.51	(0.43-0.60)	<0.001	
Q5	425/989	(43.0)	0.37	(0.30-0.46)	<0.001	
Reperfusion						0.32
Q1	108/134	(80.6)	1.00	(reference)		
Q2	103/131	(78.6)	0.73	(0.39-1.37)	0.33	
Q3	131/160	(81.9)	0.95	(0.51-1.77)	0.88	
Q4	176/248	(71.0)	0.45	(0.26-0.78)	0.004	
Q5	284/435	(65.3)	0.23	(0.13-0.41)	<0.001	

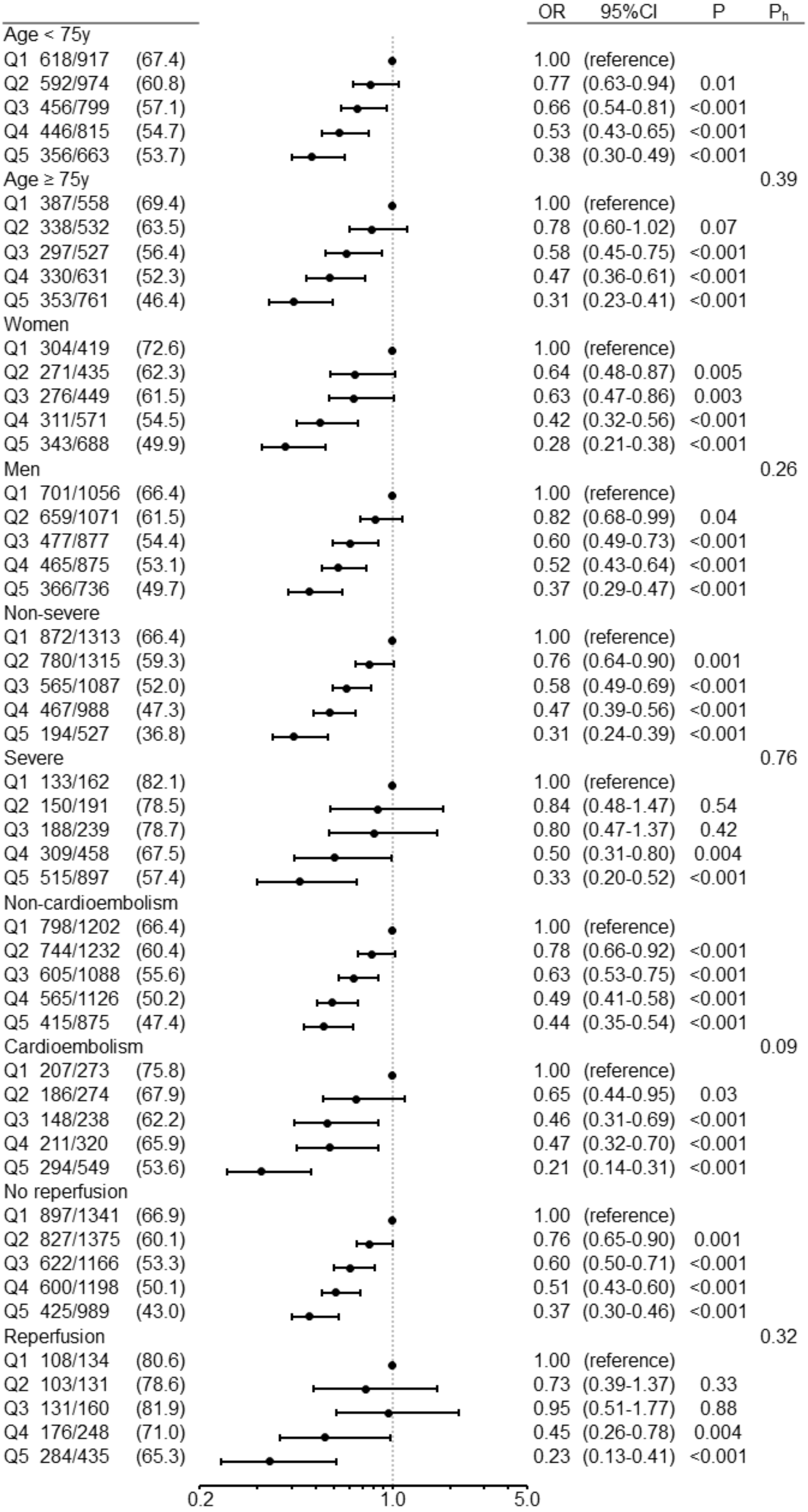

OR: odds ratio, CI: confidence interval.

OR (square) and 95% CI (horizontal bars) of neurological improvement are shown for Q1–Q4 compared to Q1 according to age (<75 y and ≥75 y), sex, stroke severity (National Institutes of Health Stroke Scale [NIHSS] score on admission: <6 and ≥6), stroke subtype (Non-cardioembolism and cardioembolism), and reperfusion therapy.

The multivariable model included the following covariates: age, sex, hypertension, diabetes mellitus, dyslipidemia, atrial fibrillation, previous stroke, body mass index, estimated glomerular filtration rate, early hospital arrival, National Institutes of Health Stroke Scale score on admission, stroke subtype, reperfusion therapy, acute infections, and C-reactive protein level.
