# Supplementary material for: Body temperature in the acute phase and clinical outcomes after acute ischemic stroke
Source: PLoS One. 2024 Jan 11;19(1):e0296639. doi: 10.1371/journal.pone.0296639 (PMC10783745; doi:10.1371/journal.pone.0296639)
Supplement: S4 Fig — OR: odds ratio, CI: confidence interval. OR (square) and 95% CI (horizontal bars) of poor functional outcome are shown for Q1–Q4 compared to Q1 according to age (<75 y and ≥75 y), sex, stroke severity (National Institutes of Health Stroke Scale [NIHSS] score on admission: <6 and ≥6), stroke subtype (cardioembolism and non-cardioembolism), and reperfusion therapy. The multivariable model included the following covariates: age, sex, hypertension, diabetes mellitus, dyslipidemia, atrial fibrillation, previous stroke, body mass index, estimated glomerular filtration rate, early hospital arrival, National Institutes of Health Stroke Scale score on admission, stroke subtype, reperfusion therapy, acute infections, and C-reactive protein level. (PDF) [file pone.0296639.s004.pdf]

S4 Figure. Subgroup analysis for the association between BT and poor functional outcome

			OR	95%CI	P	P_h
Age < 75y						
Q1	39/917	(4.3)	1.00	(reference)		
Q2	69/974	(7.1)	1.64	(1.07-2.50)	0.02	
Q3	90/799	(11.3)	2.21	(1.46-3.35)	<0.001	
Q4	153/815	(18.8)	3.37	(2.26-5.00)	<0.001	
Q5	309/663	(46.6)	6.60	(4.40-9.90)	<0.001	
Age ≥ 75y						0.56
Q1	66/558	(11.8)	1.00	(reference)		
Q2	82/532	(15.4)	1.14	(0.79-1.65)	0.47	
Q3	114/527	(21.6)	1.51	(1.06-2.14)	0.02	
Q4	270/631	(42.8)	2.77	(1.99-3.84)	<0.001	
Q5	563/761	(74.0)	5.46	(3.86-7.74)	<0.001	
Women						
Q1	39/419	(9.3)	1.00	(reference)		
Q2	36/435	(8.3)	0.85	(0.51-1.42)	0.54	
Q3	79/449	(17.6)	1.70	(1.09-2.66)	0.02	
Q4	177/571	(31.0)	2.77	(1.82-4.19)	<0.001	
Q5	443/688	(64.4)	6.31	(4.15-9.58)	<0.001	
Men						0.10
Q1	66/1056	(6.3)	1.00	(reference)		
Q2	115/1071	(10.7)	1.64	(1.17-2.28)	0.004	
Q3	125/877	(14.3)	1.96	(1.40-2.73)	<0.001	
Q4	246/875	(28.1)	3.42	(2.48-4.70)	<0.001	
Q5	429/736	(58.3)	6.13	(4.34-8.66)	<0.001	
Non-severe						
Q1	69/1313	(5.3)	1.00	(reference)		
Q2	93/1315	(7.1)	1.38	(0.99-1.92)	0.06	
Q3	123/1087	(11.3)	2.10	(1.53-2.89)	<0.001	
Q4	177/988	(17.9)	3.58	(2.64-4.87)	<0.001	
Q5	192/527	(36.4)	7.52	(5.38-10.52)	<0.001	
Severe						0.77
Q1	36/162	(22.2)	1.00	(reference)		
Q2	58/191	(30.4)	1.47	(0.88-2.45)	0.14	
Q3	81/239	(33.9)	1.72	(1.06-2.79)	0.03	
Q4	246/458	(53.7)	3.39	(2.19-5.27)	<0.001	
Q5	680/897	(75.8)	8.05	(5.22-12.42)	<0.001	
Non-cardioembolism						
Q1	75/1202	(6.2)	1.00	(reference)		
Q2	118/1232	(9.6)	1.47	(1.07-2.01)	0.02	
Q3	153/1088	(14.1)	2.06	(1.52-2.80)	<0.001	
Q4	288/1126	(25.6)	3.62	(2.71-4.84)	<0.001	
Q5	465/875	(53.1)	6.84	(5.02-9.32)	<0.001	
Cardioembolism						0.70
Q1	30/273	(11.0)	1.00	(reference)		
Q2	33/274	(12.0)	1.15	(0.65-2.06)	0.63	
Q3	51/238	(21.4)	1.51	(0.86-2.64)	0.15	
Q4	135/320	(42.2)	2.44	(1.46-4.08)	<0.001	
Q5	407/549	(74.1)	5.28	(3.18-8.77)	<0.001	
Non-reperfusion						
Q1	89/1341	(6.6)	1.00	(reference)		
Q2	128/1375	(9.3)	1.39	(1.03-1.88)	0.03	
Q3	178/1166	(15.3)	2.04	(1.53-2.73)	<0.001	
Q4	329/1198	(27.5)	3.09	(2.34-4.09)	<0.001	
Q5	595/989	(60.2)	6.12	(4.54-8.24)	<0.001	
Reperfusion						0.23
Q1	16/134	(11.9)	1.00	(reference)		
Q2	23/131	(17.6)	1.32	(0.63-2.77)	0.47	
Q3	26/160	(16.3)	1.06	(0.51-2.19)	0.88	
Q4	94/248	(37.9)	2.92	(1.53-5.57)	0.001	
Q5	277/435	(63.7)	6.21	(3.29-11.72)	<0.001	

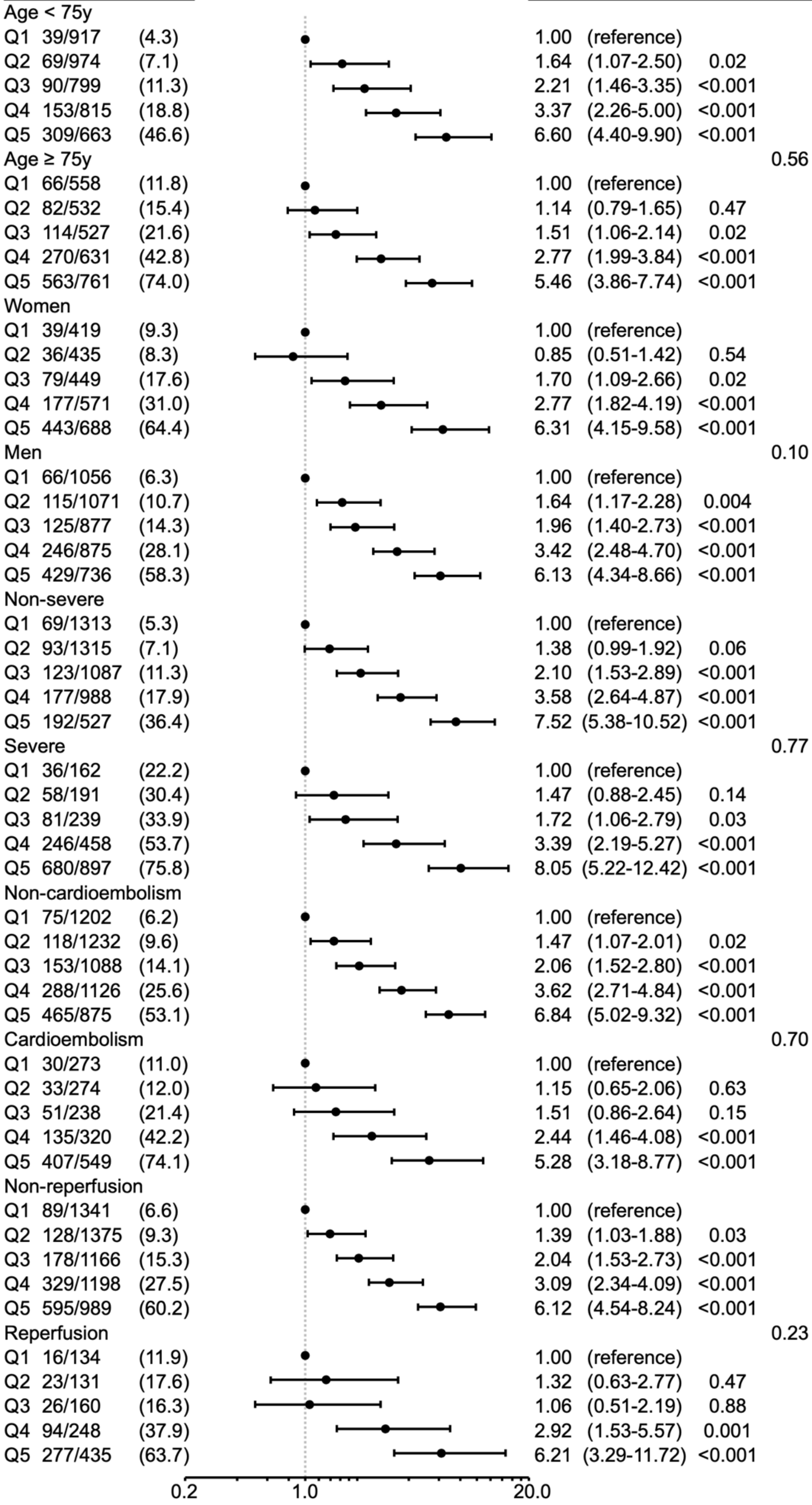

OR: odds ratio, CI: confidence interval.

OR (square) and 95% CI (horizontal bars) of poor functional outcome are shown for Q1–Q4 compared to Q1 according to age (<75 y and ≥75 y), sex, stroke severity (National Institutes of Health Stroke Scale [NIHSS] score on admission: <6 and ≥6), stroke subtype (Non-cardioembolism and cardioembolism), and reperfusion therapy.

The multivariable model included the following covariates: age, sex, hypertension, diabetes mellitus, dyslipidemia, atrial fibrillation, previous stroke, body mass index, estimated glomerular filtration rate, early hospital arrival, National Institutes of Health Stroke Scale score on admission, stroke subtype, reperfusion therapy, acute infections, and C-reactive protein level.
