# Supplementary material for: Body temperature in the acute phase and clinical outcomes after acute ischemic stroke
Source: PLoS One. 2024 Jan 11;19(1):e0296639. doi: 10.1371/journal.pone.0296639 (PMC10783745; doi:10.1371/journal.pone.0296639)
Supplement: S1 Table — BT, body temperature; OR, odds ratio; CI, confidence interval; Ptrend, P-value for trend. Q1–Q5 indicate quintiles of mean BT (°C) during the first 7 days after stroke onset. The multivariable model included the following covariates: age, sex, hypertension, diabetes mellitus, dyslipidemia, atrial fibrillation, previous stroke, body mass index, estimated glomerular filtration rate, early hospital arrival, National Institutes of Health Stroke Scale score on admission, stroke subtype, reperfusion therapy, acute infections, and C-reactive protein level. (PDF) [file pone.0296639.s005.pdf]

S1 Table. Association between BT and clinical outcomes at discharge

	Event, n	(%)	Age- and sex-adjusted			Multivariable-adjusted		
			OR	95% CI	P	OR	95% CI	P
Poor functional outcome								
Q1, 35.1–36.5 °C, n=1475	119	(8.1)	1.00	(reference)		1.00	(reference)	
Q2, 36.5–36.7 °C, n=1506	197	(13.1)	1.80	(1.41–2.29)	<0.001	1.60	(1.24–2.07)	<0.001
Q3, 36.7–36.8 °C, n=1326	246	(18.6)	2.71	(2.14–3.43)	<0.001	2.03	(1.58–2.62)	<0.001
Q4, 36.8–37.1 °C, n=1446	512	(35.4)	6.77	(5.43–8.45)	<0.001	4.02	(3.16–5.10)	<0.001
Q5, 37.1–39.1 °C, n=1424	972	(68.3)	26.15	(20.89–32.74)	<0.001	8.51	(6.59–10.99)	<0.001
P_{trend}					<0.001			<0.001
Death								
Q1, 35.1–36.5 °C, n=1475	1	(0.1)	1.00	(reference)		1.00	(reference)	
Q2, 36.5–36.7 °C, n=1506	0	(0.0)	–	–	–	–	–	–
Q3, 36.7–36.8 °C, n=1326	5	(0.4)	5.76	(0.67–49.44)	0.11	4.69	(0.54–40.74)	0.16
Q4, 36.8–37.1 °C, n=1446	8	(0.6)	7.94	(0.99–63.71)	0.05	3.73	(0.45–31.23)	0.22
Q5, 37.1–39.1 °C, n=1424	39	(2.7)	35.85	(4.89–263.12)	<0.001	6.26	(0.78–50.43)	0.09
P_{trend}					<0.001			0.008

BT, body temperature; OR, odds ratio; CI, confidence interval; P_{trend}, P-value for trend.

Q1–Q5 indicate quintiles of mean BT (°C) during the first 7 days after onset. The multivariable model included the following covariates: age, sex, hypertension, diabetes mellitus, dyslipidemia, atrial fibrillation, previous stroke, body mass index, estimated glomerular filtration rate, early hospital arrival, National Institutes of Health Stroke Scale score on admission, stroke subtype, reperfusion therapy, acute infections, and C-reactive protein level.
