# Supplementary material for: Body temperature in the acute phase and clinical outcomes after acute ischemic stroke
Source: PLoS One. 2024 Jan 11;19(1):e0296639. doi: 10.1371/journal.pone.0296639 (PMC10783745; doi:10.1371/journal.pone.0296639)
Supplement: S2 Table — BT, body temperature; OR, odds ratio; CI, confidence interval; Ptrend, P-value for trend. Q1–Q5 indicate quintiles of mean BT (°C) during the first 7 days after onset. The multivariable model included the following covariates: age, sex, hypertension, diabetes mellitus, dyslipidaemia, atrial fibrillation, previous stroke, body mass index, estimated glomerular filtration rate, early hospital arrival, National Institutes of Health Stroke Scale score on admission, stroke subtype, reperfusion therapy, acute infections, and C-reactive protein level. (PDF) [file pone.0296639.s006.pdf]

S2 Table. Association between BT, neurological courses, and poor functional outcome in patients without acute infections

	Events, n	(%)	Age- and sex-adjusted			Multivariable-adjusted		
			OR	95% CI	P	OR	95% CI	P
Neurological improvement								
Q1, 35.1–36.4 °C, n=1285	866	(67.4)	1.00	(reference)		1.00	(reference)	
Q2, 36.4–36.6 °C, n=1393	888	(63.7)	0.85	(0.72–0.99)	0.04	0.86	(0.73–1.02)	0.08
Q3, 36.6–36.8 °C, n=1176	676	(57.5)	0.65	(0.55–0.76)	<0.001	0.66	(0.56–0.79)	<0.001
Q4, 36.8–37.0 °C, n=1262	728	(57.7)	0.65	(0.55–0.76)	<0.001	0.62	(0.52–0.73)	<0.001
Q5, 37.0–39.1 °C, n=1260	668	(53.0)	0.53	(0.45–0.62)	<0.001	0.37	(0.30–0.44)	<0.001
P_{trend}					<0.001			<0.001
Neurological worsening								
Q1, 35.1–36.4 °C, n=1285	14	(1.1)	1.00	(reference)		1.00	(reference)	
Q2, 36.4–36.6 °C, n=1393	28	(2.0)	1.90	(0.99–3.62)	0.05	1.85	(0.97–3.55)	0.06
Q3, 36.6–36.8 °C, n=1176	30	(2.6)	2.43	(1.28–4.61)	0.007	2.33	(1.22–4.43)	0.01
Q4, 36.8–37.0 °C, n=1262	55	(4.4)	4.31	(2.38–7.79)	<0.001	4.28	(2.35–7.78)	<0.001
Q5, 37.0–39.1 °C, n=1260	109	(8.7)	9.09	(5.16–16.01)	<0.001	10.66	(5.92–19.17)	<0.001
P_{trend}					<0.001			<0.001
Poor functional outcome								
Q1, 35.1–36.4 °C, n=1285	79	(6.1)	1.00	(reference)		1.00	(reference)	
Q2, 36.4–36.6 °C, n=1393	128	(9.2)	1.62	(1.21–2.18)	0.001	1.43	(1.06–1.95)	0.02
Q3, 36.6–36.8 °C, n=1176	153	(13.0)	2.35	(1.76–3.13)	<0.001	1.89	(1.40–2.56)	<0.001
Q4, 36.8–37.0 °C, n=1262	259	(20.5)	4.17	(3.18–5.46)	<0.001	2.83	(2.13–3.76)	<0.001
Q5, 37.0–39.1 °C, n=1260	553	(43.9)	13.12	(10.10–17.05)	<0.001	6.21	(4.68–8.24)	<0.001
P_{trend}					<0.001			<0.001

BT, body temperature; OR, odds ratio; CI, confidence interval; P_{trend}, P-value for trend.

Q1–Q5 indicate quintiles of mean BT (°C) during the first 7 days after onset. The multivariable model included the following covariates: age, sex, hypertension, diabetes mellitus, dyslipidemia, atrial fibrillation, previous stroke, body mass index, estimated glomerular filtration rate, early hospital arrival, National Institutes of Health Stroke Scale score on admission, stroke subtype, reperfusion therapy, and C-reactive protein level.
