# Supplementary material for: Body temperature in the acute phase and clinical outcomes after acute ischemic stroke
Source: PLoS One. 2024 Jan 11;19(1):e0296639. doi: 10.1371/journal.pone.0296639 (PMC10783745; doi:10.1371/journal.pone.0296639)
Supplement: S3 Table — BT, body temperature; OR, odds ratio; CI, confidence interval; Ptrend, P-value for trend. Q1–Q5 quintiles of mean BT (°C) during the first 7 days after onset. The multivariable model included the following covariates: age, sex, hypertension, diabetes mellitus, dyslipidaemia, atrial fibrillation, previous stroke, body mass index, estimated glomerular filtration rate, early hospital arrival, National Institutes of Health Stroke Scale score on admission, stroke subtype, reperfusion therapy, acute infections, and C-reactive protein level. (PDF) [file pone.0296639.s007.pdf]

S3 Table. Association between BT, neurological courses, and poor functional outcome in patients with <37.5 °C BT

			Age- and sex-adjusted			Multivariable-adjusted		
	Events, n	(%)	OR	95% CI	P	OR	95% CI	P
Neurological improvement								
Q1, 35.1–36.4 °C, n=1026	688	(67.1)	1.00	(reference)		1.00	(reference)	
Q2, 36.4–36.5 °C, n=988	648	(65.6)	0.93	(0.78–1.12)	0.47	0.97	(0.80–1.18)	0.76
Q3, 36.6–36.7 °C, n=1004	609	(60.7)	0.75	(0.63–0.90)	0.002	0.82	(0.68–0.99)	0.04
Q4, 36.7–36.8 °C, n=968	546	(56.4)	0.62	(0.52–0.75)	<0.001	0.68	(0.56–0.82)	<0.001
Q5, 36.8–37.3 °C, n=973	510	(52.4)	0.53	(0.44–0.63)	<0.001	0.57	(0.46–0.69)	<0.001
P_{trend}					<0.001			<0.001
Neurological worsening								
Q1, 35.1–36.4 °C, n=1026	11	(1.1)	1.00	(reference)		1.00	(reference)	
Q2, 36.4–36.5 °C, n=988	23	(2.3)	2.23	(1.08–4.59)	0.03	2.13	(1.02–4.43)	0.04
Q3, 36.6–36.7 °C, n=1004	17	(1.7)	1.61	(0.75–3.46)	0.22	1.30	(0.59–2.84)	0.51
Q4, 36.7–36.8 °C, n=968	43	(4.4)	4.45	(2.28–8.69)	<0.001	3.63	(1.84–7.19)	<0.001
Q5, 36.8–37.3 °C, n=973	74	(7.6)	8.00	(4.21–15.19)	<0.001	6.26	(3.23–12.14)	<0.001
P_{trend}					<0.001			<0.001
Poor functional outcome								
Q1, 35.1–36.4 °C, n=1026	66	(6.4)	1.00	(reference)		1.00	(reference)	
Q2, 36.4–36.5 °C, n=988	89	(9.0)	1.52	(1.08–2.12)	0.02	1.32	(0.93–1.88)	0.12
Q3, 36.6–36.7 °C, n=1004	114	(11.4)	1.92	(1.39–2.65)	<0.001	1.58	(1.12–2.22)	0.009
Q4, 36.7–36.8 °C, n=968	137	(14.2)	2.52	(1.84–3.44)	<0.001	1.86	(1.33–2.60)	<0.001
Q5, 36.8–37.3 °C, n=973	255	(26.2)	5.61	(4.18–7.53)	<0.001	3.48	(2.53–4.78)	<0.001
P_{trend}					<0.001			<0.001

BT, body temperature; OR, odds ratio; CI, confidence interval; P_{trend}, P-value for trend.

Q1–Q5 quintiles of mean BT (°C) during the first 7 days after onset. The multivariable model included the following covariates: age, sex, hypertension, diabetes mellitus, dyslipidemia, atrial fibrillation, previous stroke, body mass index, estimated glomerular filtration rate, early hospital arrival, National Institutes of Health Stroke Scale score on admission, stroke subtype, reperfusion therapy, acute infections, and C-reactive protein level.
